# Evaluation on the phenotypic diversity of Calamansi (*Citrus microcarpa*) germplasm in Hainan island

**DOI:** 10.1038/s41598-021-03775-x

**Published:** 2022-01-10

**Authors:** Yong-Hui Xin, Yuan-Xin Wu, Bin Qiao, Long Su, Shang-Qian Xie, Peng Ling

**Affiliations:** 1 Key Laboratory of Genetics and Germplasm Innovation of Tropical Special Forest Trees and Ornamental Plants, Ministry of Education, Haikou, 570228 China; 2 College of Forestry, Hainan University, Haikou, 570228 China; 3 Ming Bo Scientific Technology Co., Ltd., Haikou, 571142 China

**Keywords:** Plant evolution, Plant sciences

## Abstract

Calamansi or Philippine lime (*Citrofortunella macrocarpa*) is an important crop for local economic in Hainan Island. There is no study about Calamansi germplasm evaluation and cultivar development. In this study, Calamansi data were collected from 151 of Calamansi seedling trees, and 37 phenotypic traits were analyzed to investigate their genetic diversities. The cluster analysis and principal component analysis were conducted aiming to provide a theoretical basis for the Calamansi genetic improvement. The results of the diversity analysis revealed: (1) the diversity indexes for qualitative traits were ranged from 0.46–1.39, and the traits with the highest genetic diversity level were fruit shaped and pulp colored (H′ > 1.20); and the diversity indexes for quantitative traits ranged from 0.67–2.10, with the exception of a lower in fruit juice rate (1.08) and lower in number of petals (0.67). (2) The clustering analysis of phenotypic traits have arranged the samples into 4 categories: the first group characterized by fewer flesh Segment number per fruit (SNF) and more Oil cell number (OCN); the second group had 7 samples, all characterized with larger Crown breadth (CB), higher Yield per tree (YPT), the lager leaf, the higher Ascorbic acid (AA), and less Seed number per fruit (SNPF); the third group had 25 samples characterized by smaller Tree foot diameter (TFD),smaller Fruit shape index (FSI) and higher Total soluble solids (TSS) contain; the fourth group had 87 samples, they were characterized by shorter Petiole length (PEL), larger fruit, higher Juice ratio (JR), multiple Stamen number (SN) and longer Pistil length (PIL). (3) The principal component analysis showed the values of the first 9 major components characteristic vectors were all greater than 3, the cumulative contribution rate reach 72.20%, including the traits of single fruit weight, fruit diameter, tree height, tree canopy width etc. Finally, based on the comprehensive main component value of all samples, the Calamansi individuals with higher testing scores were selected for further observation. This study concludes that Calamansi seedling populations in the Hainan Island holds great genetic diversity in varies traits, and can be useful for the Calamansi variety improvements.

## Introduction

Calamansi (*Citrus microcarpa*) or Philippine lime, is an important local economic crop in Hainan China. It originated in Southeast Asia, mainly grow in Southeast Asia and tropical regions of China, and it had a long history of cultivation in Hainan Island. Calamansi fruit is rich in vitamins C, aromatic oils, carotenoids and other natural substances which have lots of health benefits for human, such as beneficial effects for human eyes, good for treating cough, asthma, high blood pressure and preventing arteriosclerosis etc^1–3^. Calamansi fruit had a fine texture and sour taste. Calamansi juice is widely loved as a delicious fresh condiment. However, the commercially cultivated Calamansi were mostly seedling trees, and their genetic diversity and improvement had not been studied, which causing a series of problems such as no stable commodity supply period and uneven fruit quality. Hainan island is the main growing area of Calamansi in China. The investigations and evaluations of the germplasm of Calamansi in Hainan Island hold great significance for Calamansi genetic improvement with fruit quality.

Phenotypic traits were intuitive manifestations of the quality of germplasm resources and an important indicator of genetic improvement. The diversity of phenotypic traits was the comprehensive performance of the genetic diversity of germplasm and environmental effects. It had both stability and variability^4^. The evaluation of germplasm phenotypic traits were important for identifying traits with high economic value and high ecological value, and could help to identify excellent genetic resources for the subsequent variety development^5,6^.

At present, the methods frequently used for phenotypic trait evaluation included diversity analysis, correlation analysis, cluster analysis and principal component analysis^7,8^. Wang^4^ used principal component analysis, cluster analysis and other methods to analyze 312 safflower germplasm materials from all over the world, and separated them into 7 groups, provided scientific basis for the effective use of safflower germplasm for breeding of new varieties; Zhao^9^ used the same methods and analyzed the 20 traits of 257 Jerusalem artichoke germplasm, and separated Jerusalem artichoke germplasm into 5 categories, which provided reference for utilization of Jerusalem artichoke germplasm resources. Currently, most researches on Calamansi were mainly focused on the processing and utilization of Calamansi fruit-related products. Fang^10^ used Calamansi juice and bread as the main raw materials to ferment and prepare Calamansi kvass drink; Sun^11^ used Calamansi as raw material, extracted pigments and studied its physical and chemical properties which broadened the production market for Calamansi related products. However, the research and evaluation of the phenotypic traits of Calamansi was relatively lagging behind. In this study, diversity analysis, correlation analysis, cluster analysis and principal component analysis were conducted to evaluate the phenotypic traits of Calamansi germplasm resources from Hainan Island. This study is intended to lay the foundation for breed selection of Calamansi in Hainan Island.

## Results

### Genetic diversity

**Figure 1:**
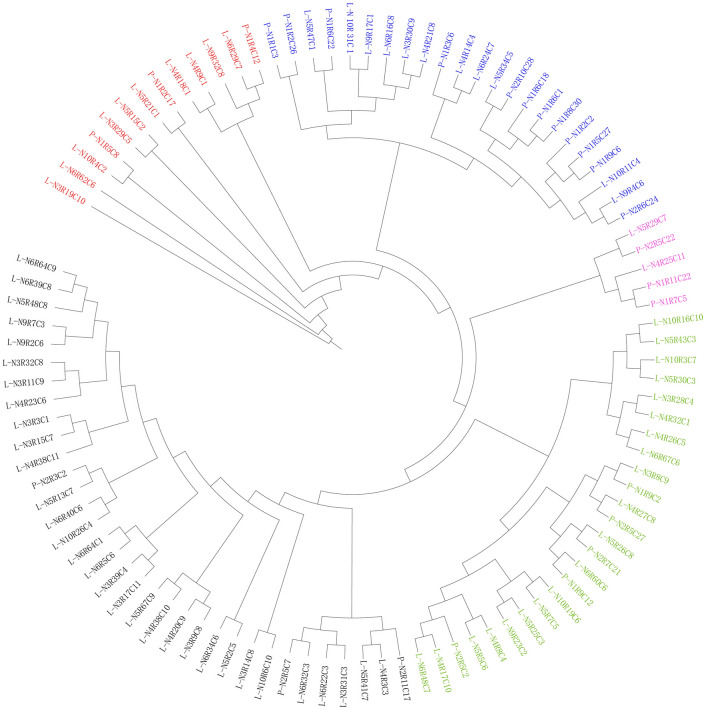
Population with 100 individuals were divided into 5 groups, separated by different colors. Phylogenetic tree of Calamansi constructed by SNPs genotypes extracted from a seedling population with 100 individual’s (figure is generated by iTOL software, Version iTOL 6.0, https://itol.embl.de/).

A total of 8,511,230 SNP loci were obtained and based on these SNP loci the phylogenetic tree was constructed by using Phylip software. The phylogenetic tree showed that those 100 individuals can be divided into 5 groups. Among them, most of them were related to each other in different level, except 2 individuals (L-N6R62C6 and L-N3R19C10) presented a very simple relationship to their common ancestor (Fig. 1). The result indicated that the current existing Calamansi populations in Hainan Island have quite high levels the genetic diversity, despite of high level of polyembryony nature of the Calamansi seeds.

### Phenotypic traits diversity

#### Quality traits

The names, abbreviations and units of all traits are shown in Table 1, and the detailed scoring criteria are shown in Supplementary Table 1. The 8 quality traits were divided into 39 levels (Table 2), 34 of which have the frequency ranged from 0.66 to 84.11% of samples distributions. There were 5 traits have weak frequency of distribution, they were TGV (Tree growth vigor), OFS (Oval fruit shape), DCFBS (Deep concave fruit base shape), DCFTS (deep concave fruit top shape), and CFTS (convex fruit top shape). There were 9 traits with an effective percentage less than 5%, only a few individuals in the population exhibited their phenotypes, including TP (Tree performance), LB (Leaf base), OFS (Oblate fruit shape), ObvFS (Obviate fruit shape), PFS (pyriform fruit shape), LNFBS (long neck fruit base shape), FFBS (flat fruit base shape), NCFBS (neck collar fruit base shape) and RFTS (round Fruit top shape). Traits with an effective percentage greater than 80% include DTP (draped Tree performance) and LNFBS (long neck fruit base shape), indicating these traits are relatively stable.

**Table 1:** Traits abbreviations and units.

Traits name	Abbreviations	Unit	Traits name	Abbreviations	Unit
Tree shape	TS	–	Petiole length	PEL	Mm
Tree growth vigor	TGV	–	Leaf lamina length	LLL	Mm
Tree performance	TP	–	Leaf lamina width	LLW	Mm
Leaf base	LB	–	Leaf shape index	LSI	–
Fruit shape	FS	–	Fruit weight	FW	G
Fruit top shape	FTS	–	Fruit diameter	FD	Mm
Fruit base shape	FBS	–	Fruit length	FL	Mm
Pulp color	PC	–	Total soluble solids	TSS	%
Tree foot diameter	TFD	Mm	Titratable acidity	TA	%
Tree height/crown width	TH/CB	–	Total soluble solids/titratable acidity	TSS/TA	–
Branch width	BW	Mm	Ascorbic acid	AA	mg/100 mg
Branch node length	BNL	Mm	Segment number per fruit	SNF	Number
Yield per tree	YPT	Number	Seed number per fruit	SNPF	Number
Tree height	TH	Cm	Fruit shape index	FSI	–
Crown breadth	CB	Cm	Petal number	PN	Number
Juice ratio	JR	%	Stamen number	SN	Number
Peel thickness	PT	Mm	Pistil length	PIL	Mm
Petal length	PL	Mm	Oil cell number	OCN	Number
Petal width	PW	Mm

**Table 2:** Diversity analysis of qualitative traits.

Traits	Classification and frequency(%)								Diversity index *H*′
	1	2	3	4	5	6	7	8	
TS	9.27	84.11	6.62	–	–	–	–	–	0.47
TP	23.00	68.33	8.67	–	–	–	–	–	0.69
TGV	0.00	23.33	76.67	–	–	–	–	–	0.46
LB	69.54	29.80	0.66	–	–	–	–	–	0.62
FS	0.66	12.58	28.48	0.00	0.66	14.57	3.31	39.74	1.39
FBS	0.66	80.79	14.57	1.32	1.99	0.00	0.66	–	0.90
FTS	0.00	59.60	32.45	1.32	0.00	6.62	–	–	0.63
PC	5.30	17.51	33.63	37.23	6.33	–	–	–	1.37

“–”: this item does not exist.

The Shannon–Wiener diversity index (*H*′) showed different traits had range between 0.46–1.39. These traits included Fruit shape (FS) and Pulp color (PC) (*H*′ > 1.20), consider being high genetic diversity^4^. The traits included Tree shape (TS) and Tree performance (TP) with lower genetic diversity (*H*′ < 0.60). The total value of these 8 quality traits diversity were 6.53; there were 4 types of fruit traits with diversity value of 4.29, which accounting for 65.7% of the total traits diversity value.

### Quantitative traits

Among 29 quantitative traits, the traits like Fruit weight (FW) had the range between 6.15 to 17.02 g, Seed number per fruit (SNPF) ranged between 3 to 11, and the Peel thickness (PT) ranged between 0.66 to 1.52 mm. The values of all the quantitative traits were indicated in Table 3, the coefficient of variation of all traits were distributed between 3.09 and 44.00%. The trait with largest variation was Yield per tree (YPT) (CV > 40%)^4^ indicating this trait had rich breeding potential. There were 14 traits with small variation (CV < 10%), including Branch width(BW) (6.51%), Branch node length (BNL) (8.26%), Leaf lamina length (LLL) (6.76%), Leaf lamina width (LLW) (7.50%), Leaf shape index (LSI) (4.54%), Fruit diameter (FD) (3.86%), Fruit length (FL) (5.60%), Fruit shape index (FSI) (4.05%), Total soluble solids (TSS) (5.18%), Titratable acidity (TA) (8.16%), Total soluble solids/Titratable acidity (TSS/TA) (8.34%), Segment number per fruit (SNF) (6.64%), Peel thickness (PT) (9.57%) and Petal number (PN) (3.09%). These results indicated that these traits hold relatively good genetic stability.

**Table 3:** Diversity analysis of quantitative traits.

Traits	Minimum	Maximum	Mean	SD	CV	Diversity index H′
TH	122.00	332.50	205.41	35.24	17.16	2.08
CB	165.73	361.78	247.85	40.82	16.47	2.04
TH/CB	0.90	1.69	1.22	0.16	13.52	1.97
TFD	14.61	119.06	73.28	17.81	24.30	2.04
BW	2.21	3.05	2.59	0.17	6.51	2.08
BNL	8.10	13.53	11.16	0.92	8.26	1.99
YPT	25.00	690.00	234.52	103.19	44.00	1.96
PEL	6.47	23.83	9.49	1.60	16.89	1.76
LLL	47.35	66.25	57.37	3.88	6.76	2.10
LLW	22.71	36.90	27.95	2.10	7.50	2.01
LSI	1.53	2.33	2.06	0.09	4.54	1.98
FW	6.15	17.02	12.54	1.57	12.48	2.03
FD	23.97	30.57	28.26	1.09	3.86	2.00
FL	23.47	32.00	28.88	1.62	5.60	2.03
FSI	0.91	1.16	1.02	0.04	4.05	2.05
AA	19.84	44.39	32.29	3.62	11.20	1.97
TSS	6.69	8.94	7.56	0.39	5.18	2.05
TA	6.04	10.44	8.10	0.70	8.61	2.00
TSS/TA	0.75	1.17	0.94	0.08	8.34	2.08
SNF	5.25	7.50	6.42	0.43	6.64	2.00
SNPF	3.92	10.50	7.17	1.14	15.87	2.03
JR	0.21	0.55	0.45	0.08	18.56	1.08
PT	0.66	1.52	1.22	0.12	9.57	2.01
OCN	15.72	30.56	21.10	2.97	14.07	2.05
PN	3.67	5.50	4.99	0.15	3.09	0.67
PL	7.04	16.87	11.07	1.64	14.82	2.08
PW	2.35	4.81	3.72	0.51	13.82	2.07
SN	0.00	28.00	21.85	2.19	10.02	1.50
PIL	3.12	8.92	6.39	1.26	19.71	1.99

The Shannon–Wiener diversity indexes (*H*′) of 29 quantitative traits were in the range of 0.67–2.10, traits like Juice ratio (JR) (1.08), Petal number (PN) (0.67)and others with lower indexes, indicated that the phenotypic variants of these traits were relatively small, or the distribution of each phenotype was uneven. In this study, except the Juice ratio (JR) and Petal number (PN) these two traits had relatively lower diversity index (*H*′), other traits all had *H*′ greater than 1.2, reflecting the rich phenotypes of these traits, and the distribution of each phenotype was relatively uniform.

### Correlation analysis of quantitative traits

**Figure 2:**
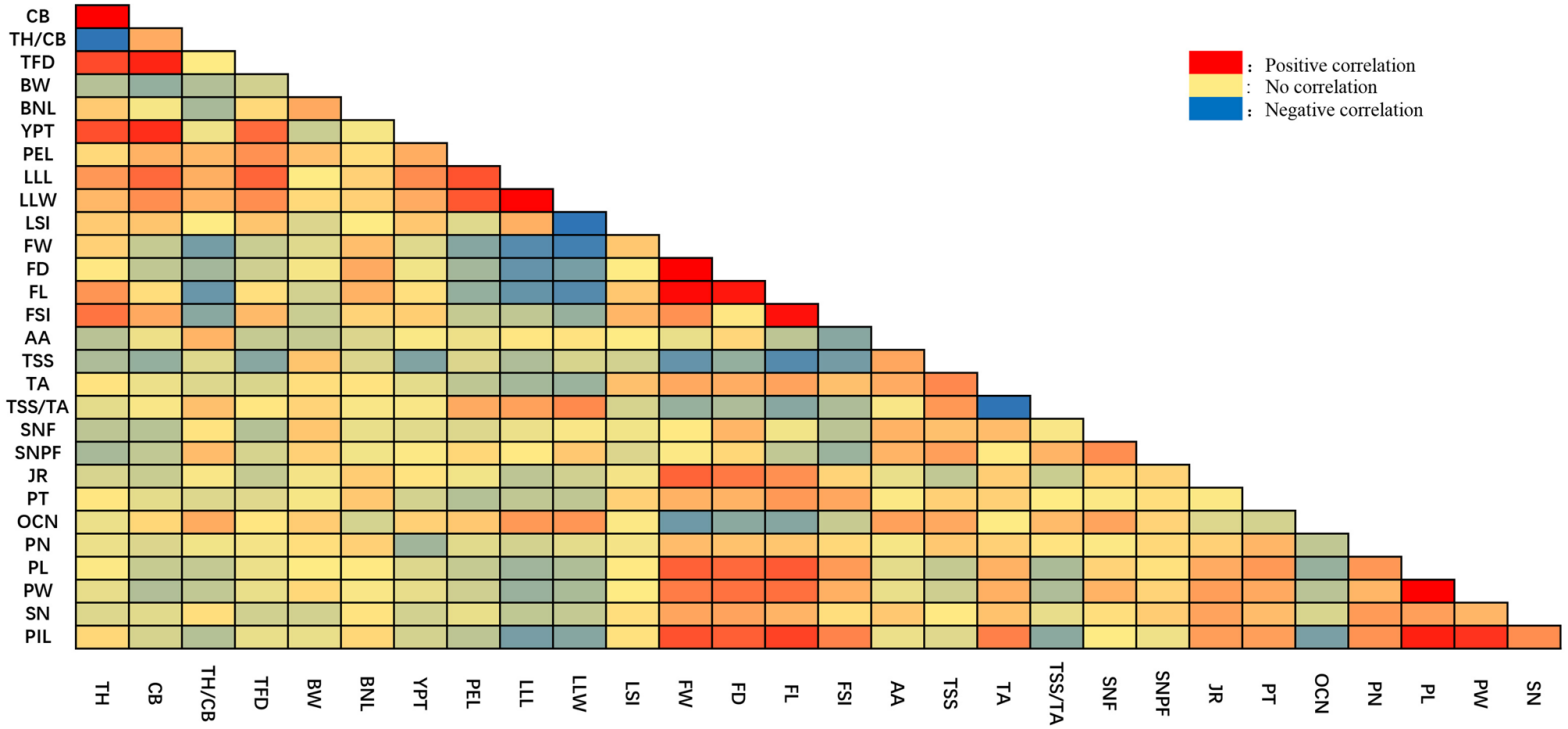
Correlation of quantitative traits among individuals. The blue area indicates a negative correlation between the two traits, and the red area indicates a positive correlation between the two traits. The darker the color the higher the level of correlation (figure is generated by EXCEL software, Version Microsoft Office 2020, https://www.office.com/).

Correlation analysis of quantitative traits showed a total of 149 pairs of traits were significantly correlated, of which 84 pairs were positively correlated and 65 pairs were negative correlated (Supplementary Table 2). Among them, like Tree Foot diameter (TFD), Branch node length (BNL), Tree height (TH), Crown breadth (CB), Petiole length (PEL), Leaf lamina length (LLL), Leaf lamina width (LLW) and were significantly correlated with each other. Fruit traits, like Fruit weight (FW), Fruit diameter (FD), Fruit length (FL), Total soluble solids (TSS), Oil cell number (OCN) and Pistil length (PIL), were also significantly correlated with other. The important trait, like Seed number per fruit (SNPF) were found negatively correlated with Tree height (TH), Crown breadth (CB), Fruit length (FL), Leaf shape index (LSI) at significant level, while positively correlated with traits like Ascorbic acid (AA), Stamen number (SN), Total soluble solids (TSS) and Total soluble solids/Titratable acidity (TSS/TA) at significant level (Fig. 2).

#### Cluster analysis

**Figure 3:**
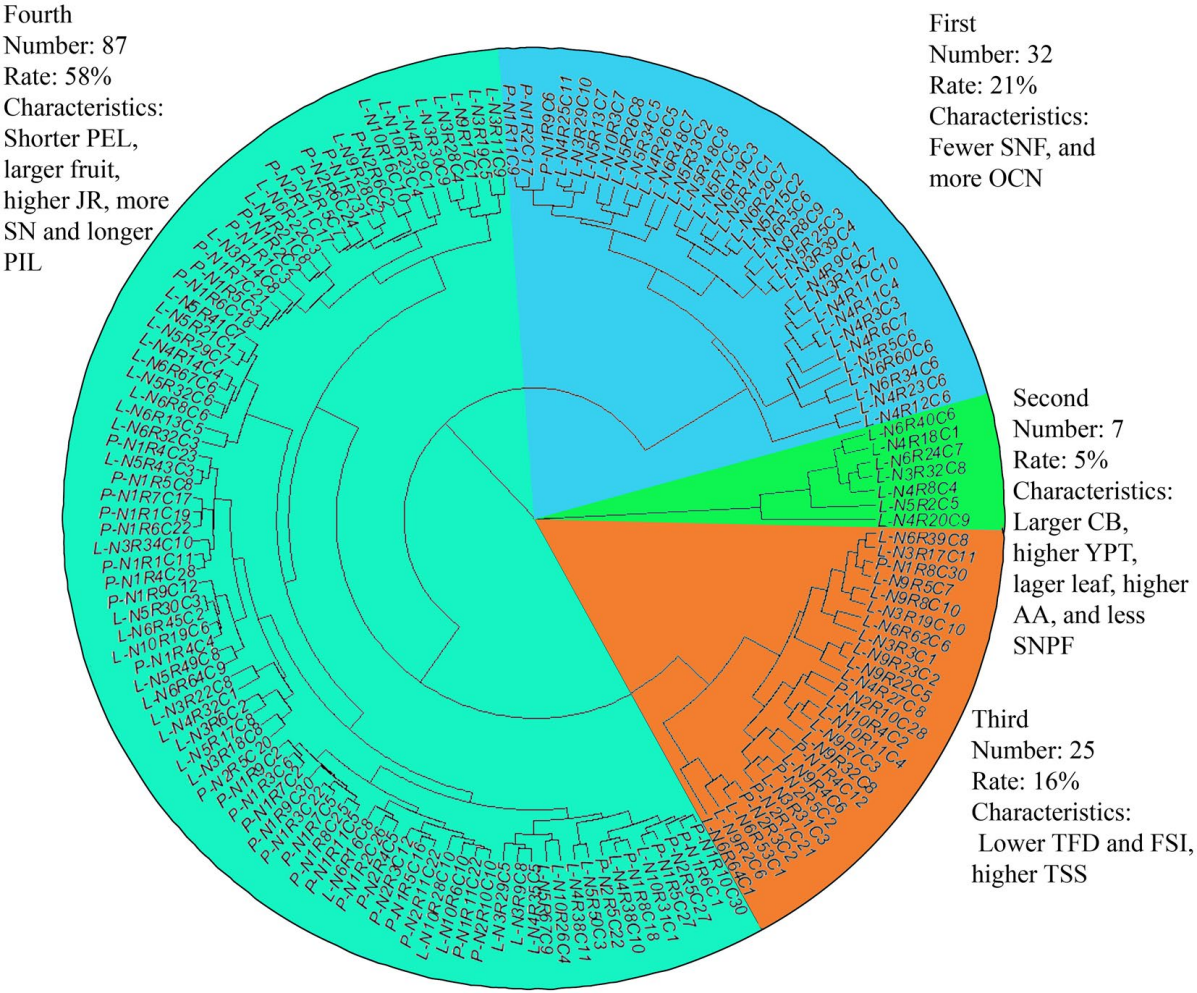
Sample cluster map. The figure shows the cluster analysis results of 151 individuals based on phenotypic traits. The results show that the populations were divided into 4 categories, which was indicated by blue, green, orange and dark green colors The number, proportion and characteristics of each category were showed in the figure (figure is generated by R software, Version R 4.1.1, https://www.r-project.org/).

The Ward method was used for conducting cluster analysis of 29 quantitative traits of the 151 individuals. The 151 individuals were divided into 4 categories (Fig. 3). A statistical analysis resulted in 4 groups: the first group containing 32 individuals; the main characteristics of this group were: fewer flesh segment number per fruit (SNF) and more oil cell number (OCN) in the fruit peel; the second group include 7 individuals, the main characteristics of this group were: larger crown breadth (CB), higher yield per tree (YPT), the lager leaf, the higher ascorbic acid (AA) and less seed number per fruit (SNPF); there were 25 individuals in the third group, the main characteristics of this group were: smaller tree foot diameter (TFD),smaller fruit shape index (FSI) and higher total soluble solids (TSS); the fourth group had 87 individuals, and characterized by shorter petiole length (PEL), larger fruit, higher Juice ratio (JR), multiple stamen number (SN) and longer pistil length.

#### Principal component analysis and comprehensive evaluation

**Figure 4:**
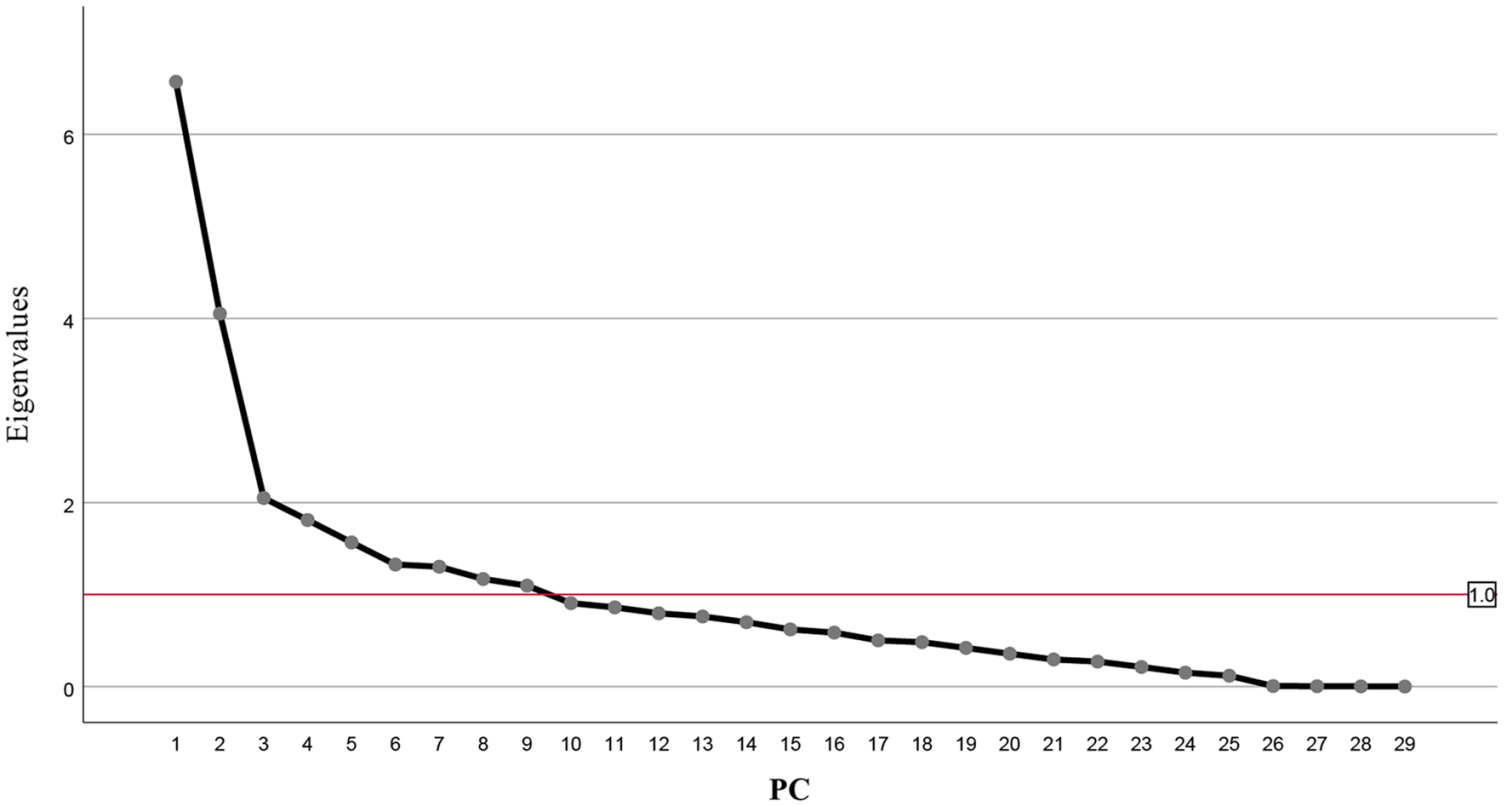
The principle component analysis showed eigenvalues of the first 9 principal components were greater than 1. The first 9 principal components had the cumulative contribution rate reach 72.20%, indicating the first 9 principal components represent most of the trait information from the phenotypic traits of Calamansi (figure is generated by SPSS software, Version SPSS 25.0, https://www.ibm.com/support/pages/node/589145).

In this study, principal component analysis was performed on 29 quantitative traits. Among the 29 quantitative traits, the eigenvalues of the first 9 principal components were greater than 1 (Fig. 4), and the cumulative contribution rate reach 72.20%, indicating that the first 9 principal components can represent most of the trait information about the 27 phenotypic traits of Calamansi (Table 4).

**Table 4:** Principal component analysis of quantitative traits.

Traits	PC1	PC2	PC3	PC4	PC5	PC6	PC7	PC8	PC9
TH	− 0.042	0.810	− 0.180	− 0.142	0.232	0.078	0.169	0.272	− 0.126
CB	− 0.339	0.752	− 0.185	0.137	− 0.127	0.142	0.222	0.083	− 0.019
TS/CB	− 0.395	− 0.151	0.033	0.369	− 0.491	0.072	0.050	− 0.284	0.156
TFD	− 0.284	0.691	0.034	0.021	0.015	0.025	0.287	− 0.091	0.206
BW	− 0.079	− 0.135	0.180	− 0.158	0.669	− 0.031	− 0.174	− 0.298	0.254
BNL	0.086	0.249	0.234	− 0.098	0.435	0.520	− 0.161	− 0.140	0.115
YPT	− 0.259	0.603	− 0.051	0.367	0.088	− 0.077	− 0.022	0.255	0.059
PEL	− 0.470	0.205	0.356	0.138	0.088	0.013	0.099	− 0.353	0.034
LLL	− 0.689	0.382	0.164	0.138	0.037	0.042	0.088	− 0.080	0.225
LLW	− 0.678	0.188	0.420	0.100	0.175	0.003	0.300	0.012	− 0.226
LSI	0.097	0.240	− 0.429	0.042	− 0.214	0.059	− 0.329	− 0.127	0.670
FW	0.776	0.202	0.177	0.219	0.019	0.210	− 0.205	0.111	− 0.009
FD	0.687	0.079	0.259	0.363	0.140	0.285	− 0.060	0.204	0.059
FL	0.774	0.450	0.039	0.049	0.036	0.054	− 0.095	0.091	− 0.063
FSI	0.415	0.564	− 0.192	− 0.279	− 0.091	− 0.183	− 0.081	− 0.066	− 0.140
AA	− 0.110	− 0.283	− 0.303	0.425	− 0.019	0.330	0.254	0.188	0.016
TSS	− 0.194	− 0.550	− 0.381	− 0.299	0.246	0.270	0.178	0.090	− 0.059
TA	0.440	− 0.130	− 0.653	0.218	0.316	0.105	0.207	− 0.291	− 0.115
TSS/TA	− 0.558	− 0.226	0.423	− 0.397	− 0.186	0.072	− 0.116	0.358	0.085
SNF	0.059	− 0.465	− 0.020	0.373	0.293	− 0.205	0.042	0.256	0.147
SNPF	− 0.089	− 0.484	0.246	0.290	0.092	0.117	0.226	0.225	0.235
JR	0.425	− 0.060	0.305	0.370	− 0.077	0.037	− 0.369	− 0.126	− 0.306
PT	0.371	− 0.064	− 0.065	− 0.381	− 0.093	0.142	0.078	0.355	0.263
OCN	− 0.571	− 0.234	− 0.316	0.172	0.117	− 0.253	− 0.028	0.017	− 0.085
PN	0.304	− 0.169	0.183	− 0.357	− 0.126	0.312	0.375	− 0.278	0.029
PL	0.738	0.023	0.232	− 0.014	− 0.013	− 0.315	0.358	− 0.013	0.181
PW	0.681	− 0.065	0.214	0.071	0.086	− 0.447	0.281	− 0.009	0.209
SN	0.367	− 0.181	0.066	0.098	− 0.392	0.331	0.180	− 0.105	− 0.073
PIL	0.824	0.072	0.037	− 0.051	− 0.023	− 0.072	0.278	− 0.089	− 0.017
Eigenvalue (E)	6.570	4.051	2.048	1.810	1.566	1.326	1.302	1.169	1.097
Contribution (%)	22.655	13.969	7.064	6.240	5.400	4.573	4.491	4.031	3.783
Cumulative Percent (%)	22.655	36.624	43.687	49.927	55.327	59.900	64.390	68.421	72.204

The PC1 had the largest contribution rate of 22.66%. The larger characteristic vectors were Fruit weight (FW), Fruit length (FL) and Pistil length (PIL), indicated that the first principal component was mainly affected by traits related to pistil length and fruit size. The contribution rate of the PC2 was 12.97%, and the larger eigenvector values were Tree height (TH) and Crown breadth (CB), indicated that the second principal component was mainly affected by the traits related to the tree. The contribution rate of the PC3 was 7.06%, and the larger eigenvector value was the Titratable acidity (TA), indicated that the third principal component was mainly affected by the titratable acid content. The contribution rate of the PC4 was 6.24%, and the larger eigenvector value was Ascorbic acid (AA). The contribution rate of the PC5 was 5.40%, and the trait with the largest eigenvector value was Branch width (BW). The contribution rate of the PC6 was 4.57%, and the trait with the largest eigenvector value was Branch node length (BNL). The contribution rate of the PC7 was 4.49%, and the traits with the largest eigenvector values were Petal number (PN) and Juice ratio (JR). The contribution rate of the PC8 was 4.03%, and the traits with the largest eigenvector values were Petiole length (PEL). The contribution rate of the PC9 was 3.78%, and the trait with the largest eigenvector value was Leaf shape index (LSI).

Comprehensive evaluation results showed that the comprehensive PC values of all samples were distributed between 38.63 and 97.41 (Supplementary Table 3), with a median of 68.02. There were 33 samples with comprehensive PC values greater than the median (Table 5), occupying all samples 21.85% of the total value.

**Table 5:** Principal component values of 33 samples.

Sample ID	F1	F2	F3	F4	F5	F6	F7	F8	F9	F
L-N5R2C5	− 304.11	951.20	− 128.02	246.84	99.26	66.99	178.71	251.41	13.02	97.41
L-N6R40C6	− 283.18	856.75	− 104.29	237.67	88.04	58.49	164.87	220.40	25.73	87.68
L-N4R20C9	− 268.57	831.38	− 109.73	234.44	84.80	64.91	157.97	224.49	14.11	86.39
L-N4R18C1	− 255.92	811.74	− 99.26	203.19	81.81	69.71	168.93	198.50	20.40	85.05
L-N5R5C6	− 304.41	839.58	− 101.05	306.83	100.08	29.88	122.21	253.05	33.63	84.06
L-N6R24C7	− 281.11	823.62	− 104.12	238.00	83.60	61.09	155.16	215.59	23.31	82.71
L-N4R8C4	− 264.90	791.44	− 96.18	236.81	79.78	56.37	146.46	212.17	22.71	81.40
L-N3R32C8	− 281.31	812.43	− 103.10	249.60	72.38	55.01	152.08	211.32	26.79	80.84
L-N6R60C6	− 241.13	763.69	− 104.22	172.98	69.11	76.94	171.09	181.75	7.58	78.03
L-N5R15C2	− 235.20	738.22	− 93.22	170.92	65.56	80.95	173.20	165.96	16.14	76.24
L-N6R34C6	− 218.11	711.35	− 91.23	170.66	71.12	70.89	158.19	172.21	11.11	75.71
L-N3R15C7	− 250.63	754.71	− 105.46	189.08	61.45	78.30	164.78	182.85	9.33	75.02
L-N4R6C7	− 240.95	728.20	− 89.01	195.65	71.55	66.93	154.21	178.13	20.92	74.88
L-N5R7C5	− 232.80	724.32	− 94.75	159.55	64.25	77.52	166.74	161.21	12.57	73.18
L-N6R29C7	− 222.45	694.62	− 90.16	190.43	69.42	62.79	144.85	179.07	11.91	72.94
L-N4R17C10	− 263.34	746.01	− 99.00	214.97	65.64	67.03	154.40	186.55	19.13	72.76
L-N4R3C3	− 211.37	682.59	− 82.07	155.47	70.96	72.49	156.07	158.11	15.31	72.48
L-N5R48C8	− 228.83	711.13	− 94.85	163.46	66.20	73.30	163.87	163.94	12.64	72.37
L-N4R9C1	− 237.14	711.43	− 94.10	187.73	65.94	68.33	157.11	170.91	18.57	72.06
L-N6R19C3	− 221.84	693.31	− 94.46	184.54	69.31	65.86	140.75	177.68	9.90	72.04
L-N4R11C4	− 231.25	700.90	− 89.98	191.44	66.97	63.06	146.57	173.50	18.03	71.87
L-N6R48C7	− 242.82	718.27	− 93.64	177.48	65.29	74.26	167.72	167.33	16.49	71.61
L-N5R34C5	− 220.54	677.76	− 72.66	179.08	66.55	62.46	148.34	153.50	22.60	70.91
L-N4R23C6	− 180.17	643.82	− 89.77	117.96	60.13	85.52	158.95	143.20	− 0.81	70.18
L-N5R26C8	− 169.77	614.46	− 76.78	140.27	65.76	66.01	142.89	146.68	10.20	69.99
L-N5R47C1	− 171.42	614.78	− 77.28	143.97	62.33	67.58	143.00	146.33	10.47	69.74
L-N5R25C3	− 217.80	668.99	− 82.65	173.07	71.21	62.62	146.01	164.47	14.11	69.50
L-N5R33C2	− 212.85	669.55	− 88.10	159.06	60.09	72.26	156.07	154.70	11.08	69.22
L-N4R26C5	− 231.20	694.80	− 91.08	161.52	59.82	72.95	163.14	155.55	15.08	69.06
L-N3R39C4	− 229.97	671.25	− 79.75	201.18	65.15	55.44	136.79	171.89	23.11	68.59
L-N4R12C6	− 213.49	658.58	− 78.61	159.92	82.98	52.33	139.81	161.65	18.40	68.42
L-N6R32C3	− 206.03	653.62	− 80.30	154.49	57.49	72.88	151.39	149.10	12.26	68.31
P-N1R6C18	− 172.04	603.65	− 74.71	149.23	63.49	63.30	138.06	147.87	10.42	68.26

## Discussion

Phenotypic traits are the reflection of the comprehensive effects of the plant genotype and the environmental effects. Phenotype is an important manifestation of genetic variation, and it can directly indicate the abundance of specific genes. Phenotype is the basis for the germplasm innovative and variety improvement^14^. In this study, the phenotypic traits of 151 Calamansi samples from Hainan Island were statistically analyzed and evaluated. The results showed that the diversity indexes of the Calamansi phenotypic traits ranged from 0.46 to 2.10, with an average value of 1.72, indicating there were rich genetic diversity among the phenotypic traits of seedling Calamansi, and the Calamansi population could be selected and used in Calamansi genetic improvements. The coefficient of variation in genetic parameters could reflect the degree of dispersion of a trait to a certain extent. The larger the coefficient of variation, the higher the degree of dispersion^15,16^. In general, if the coefficient of variation was greater than 10%, indicating that the trait varies among different germplasm individuals were diversified^17^. The coefficient of variation of 14 phenotypic traits, Branch width (BW), Branch node length (BNL), Leaf lamina length (LLL), Leaf lamina width (LLW), Leaf shape index (LSI), Fruit diameter (FD), Fruit length (FL), Fruit shape index (FSI), Total soluble solids (TSS), Titratable acidity (TA), Total soluble solids/Titratable acidity (TSS/TA), Segment number per fruit (SNF), Peel thickness (PT) and Petal number (PN) were less than 10%, means the genetic performance was relatively stable. Among the quantitative traits, the variation coefficient of the Yield per tree (YPT) was relatively larger; others were distributed between 10.02 and 21.30. The variation of quantitative traits of Calamansi were distributed between 3.09 and 44.00%, indicated that there were large diversity in the quantitative traits among the individual samples, and implied that there was a good breeding potential in the Calamansi population studied. The cluster analysis results had separated the samples into 4 categories: (1) fewer Segment number per fruit (SNF) and more oil cell number (OCN); (2) larger crown breadth (CB), higher yield per tree (YPT), fewer seed number per fruit (SNPF); (3) lower tree foot diameter (TFD) and fruit shape index (FSI), but higher total soluble solids (TSS) and (4) higher titratable acidity (TA), shorter petiole length (PEL), larger fruit diameter (FD) and fruit length (FL), higher juice ratio (JR). This analysis could provide elite individual plant materials to support the Calamansi breeding development. This study was based on the phenotypic traits of Calamansi, using Principal component analysis (PCA), it was found that the cumulative contribution rate of the first nine principal components of Calamansi was 72.20%, which could represent most of the Calamansi, and perhaps implied that those phenotypic traits could be integrated at the same time. Through this analysis, those individuals with higher scores from comprehensive evaluation were selected. This comprehensive evaluation method had been used in the phenotyping and the classifications of other crops^18–20^. The results of this study could be used to select Calamansi individuals with outstanding traits.

In addition, this research also found that the Calamansi seeds have extremely high level of polyembryonic, but the diversity analysis of the Calamansi population resulted relative higher diversity index, and phenotypic evaluation also showed relative higher diversity among the traits analyzed. This interesting phenomenon might imply high frequency of sprout mutation existing in the Calamansi germplasm population which caused relative high genetic diversity in descendant population after multi-generation of propagation by seeds. Another possibility is that in the long history of cultivation, open-pollinated Calamansi zygotic embryos under the growth pressure, had gradually produced stronger competitive ability than the somatic embryos, and developed into complete individuals, leading to the continuous evolution of Calamansi and phenotypic diversity. Finally, in the process of data collection, it was found that harvested fruits within the commercial standards weight range (10–13 g per fruit) had about twice as more seed numbers than that of fully mature Calamansi fruits. The reasons of this phenomenon and seed number reduction mechanism were unknown at the present time.

This study investigated 151 individuals of the Calamansi germplasm resources in Hainan Island, and evaluated various phenotypic traits of cultivated Calamansi. The research provided information for the whole genome association analysis of Calamansi. The resulting data proved to be useful in the subsequent genome-wide association analysis, which built up the connection between Calamansi’s phenotype and the responsible genes.

This article is the first research to investigate the germplasm of Calamansi in Hainan Island, China. Hainan Island is a geographically isolated tropic environment. The Calamansi cultivation on the island has several hundred years history, Calamansi has under gone many generations of selections intentionally or unintentionally, the genetic variations (mutations) with advantage to their growth or beneficial to the growers were likely survived and being saved, many genetic variations were saved and cumulated resulted Calamansi’s genetic diversities in the Hainan Island. This study can reflect the genetic characteristics of Calamansi to a certain extent. Calamansi is widely distributed in many countries in Southeast Asia, and is widely used in different culture of life. In the future, all the Calamansi germplasm resources in Southeast Asia will be collected and analyzed, which can more accurately study the genetic characteristics of Calamansi and its genetic information could provide more valuable references for Calamansi breeding and cultivar improvement.

## Conclusion

In this study, the phenotypic traits of the Calamansi seedling populations in Hainan Island was first time evaluated. The study identified elite individuals for various traits, provided plant materials and data to support the subsequent Calamansi breeding operation. Since Calamansi is a widely cultivated “cash crop” in Hainan Island, it is a plant species that has important role in the local economy, especially for the farmers who only have small scale of land available. In this study, we systematically evaluated 37 phenotypic traits of the seedling populations of Clamansis, and found there were high level of genetic diversity among the Clamansis seedling populations for those traits. Existing Calmansi populations can serve as genetic resource for Calmanis variety development.

## Materials and methods

### Plant materials

**Figure 5:**
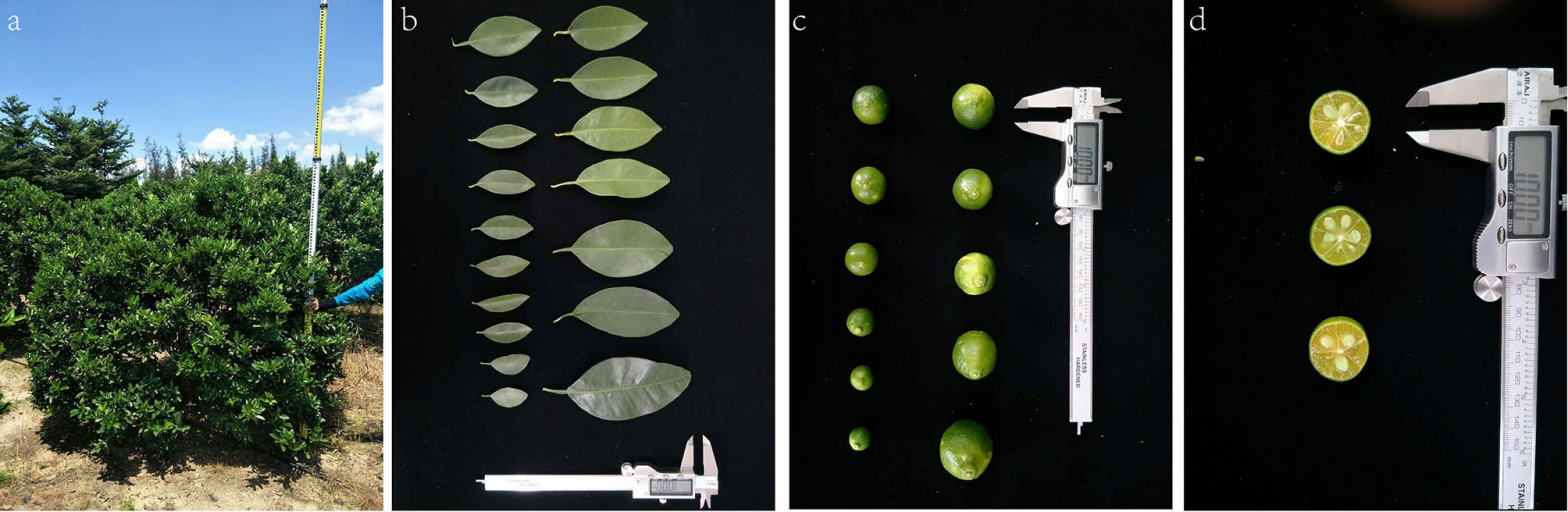
Calamanis (**a** Calamanis tree, **b** Calamanis leaf, **c** Calamanis fruit size, **d** Calamanis pulp color).

The samples of this study were collected from the planting groves of Hainan Ming Bo Scientific Technology Co., Ltd. in Quanmei, Wenchang city and Dongchang Farm, Haikou city, China (Fig. 5). The Calamansi fruit trees planted in these groves were derived from seedlings in Hainan Island. In this study, total of 151 (101 and 50) robust samples were collected from Quanmei and Dongchang Farm, respectively. The location of Quanmei in Wenchang is approximately 110° 97′ east longitude and 19° 65′ north. The location has an average annual temperature of 24.4°, an average annual sunshine of 1953.8 h, and an average annual rainfall of 1948.6 mm. The location of Dongchang is approximately 110° 36′ east longitude and 20° 01′ north latitude, with an average annual temperature of 23.8°, an average annual sunshine of 1752 h, and an average annual rainfall of 1724.5 mm. All the trees were 6–8 years old and under the same management conditions.

### Phenotypic data analysis

Various traits were evaluated from the 151 samples for 2019 and 2020 two years. The phenotypic traits were investigated, including 8 qualitative traits and 29 quantitative traits. The descriptors for citrus germplasm resources^21^ were used as standard reference (Supplementary Table 1). Among them, the quality traits of Tree shape (TS), Crown breadth (CB), Fruit shape (FS) and others were measured by comparing with standard graphs. Quantitative traits such as Tree height (TH), Leaf lamina length (LLL), Fruit weight (FW), were measured by the corresponding tower ruler, vernier caliper, and analytical balance. Traits of Total soluble solids (TSS), Titratable acidity (TA), Ascorbic acid (AA), and Juice ratio (JR) were measured by refractometer method, redox titration method, 2, 6-dichloroindophenol titration method, and physical pressing method. Each sample was repeated 6 times.

### Phenotypic diversity and statistical analysis

The data obtained from the phenotype survey were sorted and analyzed using Microsoft Office Excel 2019, and Spss 25.0. The degree of morphological diversity was expressed by Shannon–Wiener index, and the calculation formula was

$${H}^{{\prime}}=-\sum_{i=1}^{n}(PilnPi)\quad (i=1, 2, 3\ldots )$$

where *H*′ was the diversity index, ‘*n*’ was the total number of classes, and ‘*Pi*’ was the effective percentage of the material distribution frequency in the ‘*i-th*’ class of the trait. Quality traits were directly calculated according to the effective percentage of each grade. Calculated the overall average ($\overline{x}$) and standard deviation (*s*) for quantitative traits, and then from the first level < − 2* s*, the tenth level ≥ + 2* s*, and every 0.5* s* was one level. The correlation between quantitative traits was calculated using Pearson's correlation coefficient, and the principal components of quantitative traits were extracted using dimensionality reduction analysis and factor analysis (SPSS 25.0) Finally, according to the principal component weight, the comprehensive principal component value of the sample was calculated to screen the sample.

### Genetic diversity analysis

#### Sequencing of Calamansi genome and SNPs identification

In this study, after preliminary analysis the phenotypic traits of 151 Calamansi fruit tree samples, 100 fruit trees with rich phenotypic characteristics were selected and subjected to genome sequencing. The library was constructed and sequenced through the Illumina sequencing platform, and 350G raw data were obtained. After acquiring the genomic data of Calamansi, Fastp software was used to perform quality control on the sequencing data, and then quality-controlled data were compared with genomic data of *Citrus clementina*^12^ (https://www.citrusgenomedb.org/analysis/156) to obtain the corresponding comparison information. Then GATK 4.0 software was used to perform mutation screening on 100 individuals genome sequences data to obtain the corresponding gvcf files. Finally, all the gvcf files were merged into vcf files, and vcf files were further filtered to obtain SNP site of 100 individual Calamansi. Default parameters were used by all software when processing the data.

#### Construction of phylogenetic tree

The phylogenetic tree of Calamansi was constructed by Phylip software based on the neighboring method. The specific code is as follows:

- Run_pipeline.pl -Xmx1G -Xmx5G -importGuess all.filtered.snp.vcf -ExportPlugin -saveAS sequence.phy -format Phylip_Inter
- echo -e “sequences.phy\nY” > dnadist.cfg
- Dnadist < dnadist.cfg > dnadist.log
- echo -e “infile.dist\ny” > neighbor.cfg
- neighbor < neighbor.cfg > nj.log
- less infile.dist | tr ‘\n’ ‘|’ | sed ‘s/|/ /g’ | tr ‘|’ ‘\n’ > infile.dist.table
- less outtree | tr ‘\n’ ‘|’ | sed ‘s/ //g’ > outtree.nwk

Finally, the evolutionary tree was obtained by upload obtained outtree.nwk file onto the itol website online.

#### Principal component analysis and comprehensive evaluation

Principal component analysis (PCA), a statistical analysis method that converts multiple variables into a few principal components (PC1–PCn) through dimensionality reduction technology. Principal component analysis was carried out by statistical analysis software SPSS25.0. These PCs can reflect most of the information of the original variables^13^. Through the software processes, the corresponding value of each trait under each Principle component (PC) can be obtained, and the values are called the characteristic vector of the broad PC. The larger the absolute value of the trait characteristic vector, the greater the influence on the PC. One or several of the traits with the largest absolute value of the characteristic vector under the PC can be considered that this PC is controlled by these traits to a certain extent. The eigenvectors under each PC is added to obtain the eigenvalue (E) of the PC. Through the software calculation, the eigenvalue can be converted into a contribution rate. In theory, the sum of the contribution rates of all PC equals 1, which can fully explain all the information of the original variables.

According to the eigenvector matrix and standardized phenotype data, all samples were comprehensively evaluated^4^. The specific scoring formula was as follow: Fn = − 0.042 × 1 − 0.339 × 2 − 0.395 × 3 + …… + 0.681 × 27 + 0.367 × 28 + 0.824 × 29. Then the comprehensive principal component value F was calculated according to the ratio of the characteristic value corresponding to each principal component. In the calculation, the total characteristic value of the extracted principal component served as the weight to sort the comprehensive principal component value F = 0.227 × 1 + 0.140 × 2 + 0.071 × 3 + …… + 0.045 × 7 + 0.040 × 8 + 0.038 × 9.

#### Cluster analysis

The statistical analysis software SPSS25.0 was used to carry out the cluster analysis, the Ward method was used to conduct cluster analysis of 29 quantitative traits among 151 individuals. The 151 individuals were divided into 4 categories. Ward method is an alternative approach for performing cluster analysis; it looks at cluster analysis as an analysis of variance problem, instead of using distance metrics or measures of association. Ward method involves an agglomerative clustering algorithm, Ward's method starts out with n clusters of size 1 and continues until all the observations are included into one cluster. This method is most appropriate for quantitative variables cluster analysis.

### Correlation analysis of quantitative traits

Correlation analysis of 29 quantitative traits was carried out by statistical analysis software SPSS25.0 among 151 Calamansi individuals, the directions and levels of the correlation among 29 quantitative traits were indicated in (Fig. 2). Basically the correlation analysis was performed use the data from the 29 quantitative traits, Karl Pearson’s co-efficient of correlation was calculated to present the relationship between each other traits.

### Ethical approval

The collected plant materials and research activities are in accordance with the laws and regulations of Hainan Province, China.

The collection of Calamansi resources has been approved by the grove owner Ming Bo Scientific Technology Co., Ltd.

## Supplementary Information

Supplementary Tables.

## Data Availability

The data were collected by YHX and YXW. The materials were collected from the farm of Ming Bo Scientific Technology Co., Ltd.
